# Paving with Precast Concrete Made with Recycled Mixed Ceramic Aggregates: A Viable Technical Option for the Valorization of Construction and Demolition Wastes (CDW)

**DOI:** 10.3390/ma12010024

**Published:** 2018-12-21

**Authors:** Andrés Juan-Valdés, Julia García-González, Desirée Rodríguez-Robles, Manuel Ignacio Guerra-Romero, Fernando López Gayarre, Nele De Belie, Julia M. Morán-del Pozo

**Affiliations:** 1 Department of Agricultural Engineering and Sciencies, University of León, 24071 León, Spain; ignacio.guerra@unileon.es (M.I.G.-R.); julia.moran@unileon.es (J.M.M.-d.P.); 2 Department of Agriculture and Feeding, University of La Rioja, 26006 Logroño, Spain; julia.garciag@unirioja.es; 3 Department of Agronomy and Forestry Engineering, University of Extremadura, 06007 Badajoz, Spain; desireerodriguez@unex.es; 4 Department of Construction, Campus de Gijón, University of Oviedo, 33203 Gijón, Spain; gayarre@uniovi.es; 5 Magnel Laboratory for Concrete Research, Ghent University, 9052 Ghent, Belgium; Nele.DeBelie@ugent.be

**Keywords:** precast paving elements, recycled concrete, recycled mixed ceramic aggregates, mechanical, microstructural, and durability characterization

## Abstract

This research aimed to prove the feasibility of producing two types of precast elements widely used in construction, such as curbstones and paving blocks, using recycled concrete made with a 50% substitution of the natural gravel by recycled mixed aggregates with a significant ceramic content (>30%). In order to prove the quality of such mass concrete recycled precast elements, two different mixes were used: the first one was a conventional concrete mix provided by Prefabricados de Hormigón Pavimentos Páramo S.L., one of the collaborating companies in this study, and the other was a mixture in which wt 50% of the natural coarse aggregates were substituted for recycled mixed aggregates ceramic (RMAc). This recycled aggregate is a heterogeneous mixture of unbound aggregates, concrete, ceramic, etc., used as a secondary recycled aggregate and commonly produced in a lot of recycling plants in many European countries. This material was supplied by Tecnología y Reciclado S.L., the other collaborating company. Both mixtures were representative in order to establish the comparative behavior between them, taking into account that smaller percentages of replacement of the natural with recycled aggregates will also produce good results. This percentage of substitution represents a high saving of natural resources (gravel) and maintains a balanced behavior of the recycled concrete, so this new material can be considered to be a viable and reliable option for precast mass concrete paving elements. The characterization of the recycled precast elements, covering mechanical, microstructural, and durability properties, showed mostly similar behavior when compared to the analogous industrially-produced pieces made with conventional concrete.

## 1. Introduction

First world countries, those belonging to the European Union among them, have experienced a meteoric development from the industrial revolution to the present day in terms of industrial, demographic, economic, and social progress. Nevertheless, despite the economic, technical, scientific, and political advances that have been occurring recently, the idea of sustainable development remains an ideal in both developed and emerging nations.

In any case, increasing social awareness regarding the environmental problems of the planet is leading individuals to accept their role as active participants in conservation of the environment, and impelling them to follow reuse and recycling policies with ever greater eagerness. However, it is estimated that only 30% of the so-called easily recyclable wastes, namely paper, glass, and plastic, are properly deposited in their designated dumpster [1].

In addition, there is another type of waste usually generated in the urban environment which often goes unnoticed by the general public, whereas its management is a principal concern at a European level. Construction and demolition wastes (CDW) represent around a third (34.7%) of the total waste generated in Europe [2].

It is worth mentioning that management of these wastes has been strongly regulated in the last years through specific standards, with many exploring possible avenues for its recovery and use.

In order to achieve such objectives, new uses must be sought for the recycled materials originating from CDW, as a means to establish greater efficiency in the use of available resources and encourage a circular economy. European and Spanish policies, which encourage separation of the different constituents of waste, have enabled the collection of homogenous materials, which are more easily processed and can be successfully transformed into recycled aggregates with adequate properties, thus making it possible that a material traditionally considered to be a waste could regain secondary material status.

In Mediterranean nations, such as Portugal, Spain, Italy, and France, and Central European countries, where the use of ceramic materials (bricks, tiles, pavements, etc.) is a common construction practice, ceramic wastes constitute a large fraction of the CDW. For instance, Reference [3] stated that, on average, 75% of the CDW generated were of stony nature, of which more than half (54%) were ceramic materials. Therefore, the majority of Spanish CDW management plants produce recycled mixed aggregates consisting mainly of concrete, ceramics and natural aggregates, and other minority elements. Recycled aggregates originating from these heterogeneous wastes are frequently considered to be a secondary material of residual value; recycled mixed aggregates are thus commonly destined for backfilling or other low level construction applications, with very restricted use and therefore reduced opportunities for valorization. Some recent studies, however, have demonstrated that recycled aggregates can produce quality concretes inter alia [4,5,6,7,8,9].

The aim of this research work was to study the possibilities of CDW with a significant ceramic content for application as secondary material in the manufacture of non-structural prefabricated paving elements. Therefore, the mechanical, microstructural, and durability properties of the recycled concrete made with an established dosage of recycled mixed ceramic aggregates (RMAc) were evaluated against a reference concrete. On this subject, some other studies have also assessed the use of recycled aggregates from CDW in the preparation of precast concrete [10,11,12,13,14,15,16,17]. The present paper researches a complete characterization of recycled curbstones and paving blocks, focusing its attention on physical aspects that could define the durability of precast elements. This work completes others, inter alia [11,18] and is a new step in the research area, including a more exhaustive characterization of the recycled concrete, especially in those characteristics related to durability. This manuscript includes new tests of water absorption, abrasion, electrical resistivity, and freeze–thaw, four different tests that were not included in previous works and that give an accurate idea of the durability of recycled concrete when compared to control concrete. This new study gives a complete idea of the mechanical, microstructural, and durability behavior of recycled concrete, which may lead to important improvements.

In addition, the results obtained extend widely to the preparation of recycled non-structural precast concretes mixed with mixed ceramic recycled aggregates. According to data from Reference [19], the majority of recycled aggregates received in treatment plants in Spain are recycled mixed ceramic aggregates (RMAc) with more than 70% ceramic content. Survey studies, including that in Reference [19] (with more than 74 plants polled during a 3 years period), have returned results indicating that the average composition of RMAc is very similar to the recycled aggregates used in this work (shown in this manuscript in Table 2). Moreover, more than 2/3 of the polled plants produced RMAc, while only 1/3 produced only 100% concrete recycled aggregate or other types of products.

The main novelties of this work are the use of commercial recycled mixed ceramic aggregates as secondary aggregates for the production of precast concrete paving elements, and a complete and extensive characterization of these elements, including their marks according their respective European Norms (EN). Many works have dealt with these types of aggregates, but many of them did not employ commercial recycled aggregates produced in a recycling plant, so their field of real-world application is reduced. Two different mixes were used to establish the comparison: a conventional concrete mix provided by a collaborating company involved in this research, and a recycled concrete mix using commercial secondary coarse aggregates (substitution of 50% of coarse aggregates) produced in recycling plants, without any other treatment or modification, so the potential of this material can be evaluated in real conditions. Both mixtures were representative in order to establish a comparison between them, taking into account the fact replacing smaller percentages of the natural with recycled aggregates will also produce good results. In addition, the comparison between commercial concrete mixtures and recycled concrete mixtures gives an accurate idea of the potential of this new materials, offering a realistic view of the possibilities of their use.

## 2. Materials and Methods

### 2.1. Concrete Raw Materials

To manufacture of the different concrete mixtures, the following materials were employed in this research:

Commercially available blended Portland cement (CEM III/A 42.5 N/SR, Cementos Tudela Veguin, Aboño, Spain) conforming to the standards outlined in References [20,21]. Besides the environmental advantages, a blended cement including blast furnace slag was selected based on the recommendations of Reference [22]. The authors reported that sulphate-resistant CEM III/A was especially suitable for the manufacture of recycled concrete since it resulted in mixes with lower strength decline compared to conventional concrete, and due to its resistance against the greater sulphate content associated with the use of recycled aggregates. The 42.5 N strength class was chosen to match the one used by the local precast concrete company.

Tap water was used, complying with the recommendations in Reference [23].

**Figure 1:**
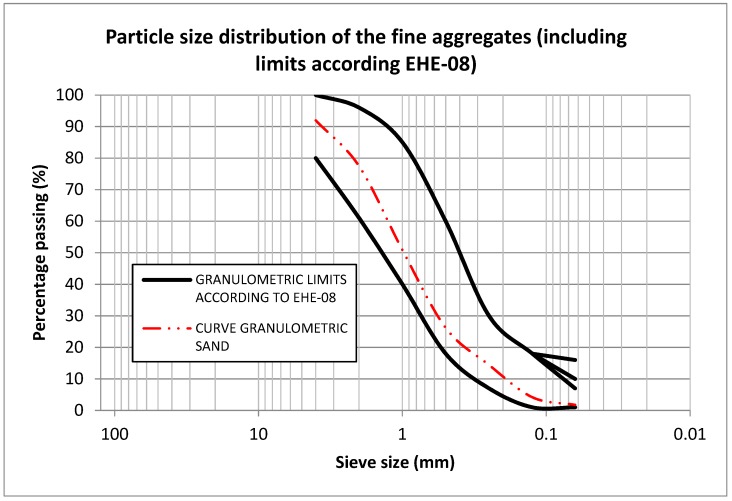
Particle size distribution of the fine aggregates used in the study, including limits according to EHE-08 (Spanish standard for concrete).

**Figure 2:**
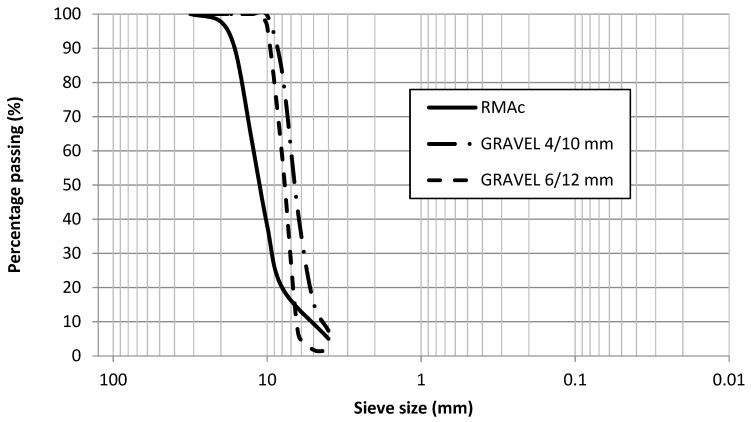
Particle size distribution of the coarse aggregates: natural (gravel) and recycled (RMAc).

Both the fine and coarse natural aggregates presented a siliceous nature, and complied with the requirements of References [23,24] establishing the properties that aggregates used in the manufacture of concrete must fulfil. In addition, they hold the CE marking of construction products (certificate number: 1035-CPR-ES033899). All natural aggregates used are commonly used in the production of precast specimens: 0/4 mm crushed sand, 0/5 mm rounded sand, 4/10 mm gravel, and 6/12 mm gravel. Figure 1 and Figure 2 display the particle size distribution accordance with Reference [25], and a comparison of the different fractions of natural and recycled aggregates.

The recycled aggregates were obtained through mechanical treatment (crushing, sieving, and removal of impurities) of the CDW in a recycling plant located in the Autonomous Community of Madrid (Spain). The aggregate characterization carried out revealed differences between the recycled aggregates and natural aggregates. In terms of physical and mechanical properties such as D/d ratio, fines content [25], flakiness index [26], or Los Angeles coefficient [27], RMAc performed similarly to the natural aggregates, and the results were within the suitable range established by Reference [23] for concrete manufacture. However, results obtained following the tests specified in Reference [28] showed variations compared to the natural aggregates. The presence of attached mortar and ceramic materials in the recycled aggregates caused a reduction in density of RMAc (2.08 Mg/m^3^) in comparison with natural aggregates (2.54 Mg/m^3^). Nonetheless, the main difference between the RMAc and the natural aggregates was water absorption, which was significantly affected by the properties of the original attached mortar, as previously stated [29,30,31,32,33]. In this case, RMAc showed a water absorption value of 8.5%, higher than the water absorption value of natural coarse aggregates at 1.2%. This increase was attributed to the presence of old mortar and clay materials [12,34,35]. Despite the commonly dry conditions used in the manufacture of precast concrete elements, the use of aggregates with high water absorption could result in a workability drawback. Hence, a technique to solve this problem was required. Previous studies using the same recycled aggregates employed in this paper have shown that the technique of pre-saturation of the recycled aggregate is a suitable method to quickly, easily, and inexpensively manufacture recycled concrete with low strength requirements and maintain a suitable workability [36]. This technique modifies the moisture state of the aggregate through partial saturation of the superficial pores of the recycled aggregates, decreasing their water absorption during the concrete mixing process, and keeping the process water-free until the cement hydration.

Finally, the composition of the recycled aggregates was determined according to Reference [37]. Table 1 and Table 2 show the properties and composition (wt %) of the non-floating components of the mixed recycled aggregate (RMAc).

The recycled aggregates were composed of the following materials: concrete and mortar, unbound natural aggregates, ceramics, asphalt, glass, gypsum, and impurities such as wood, plastic, and metal. The data showed that the predominant material was unbound natural aggregates (44.11%) followed by materials of a ceramic nature, which constituted 33.56%, and the concrete and mortar fraction (17.51%). In terms of impurities, such as glass, asphalt, wood, paper, metals, and plastic, no significant problems were expected based on the quantities obtained. However, the great content of gypsum (3.48%) had potential to generate some problems, as its incorporation could have caused expansions in the recycled concrete due to delayed formation of ettringite [38]. Nevertheless, as can be observed in the accompanying scanning electron microscope (SEM) images, such problems were not identified for the recycled mixture assessed in this paper.

### 2.2. Concrete Mix Proportions

Although some efforts have been made to define a specific mix design for recycled concrete, the common practice is based on the substitution, in terms of weight or volume, of natural aggregates by recycled ones. However, it is recognized that the greater water absorption of recycled aggregates, which is higher particularly when fine recycled aggregates are included in the replacement, is greatly responsible for the differences between recycled and conventional mixes and may cause problems in recycled concrete performance, especially if not considered during the mixing stage by means of a water compensation technique.

Ideally, recycled concrete should be able to replace the equivalent commercially available conventional concrete option. Therefore, the proportions of the conventional concrete (CC) mix used by Prefabricados de Hormigón Pavimentos Páramo S.L. (La Bañeza, Spain) in the manufacture of commercially available curb units and paving blocks was used as a model for the dosage of the recycled concrete (RC) specimens.

In order to produce recycled non-structural precast elements that can economically compete with conventional products, the cement content for both mixtures should be the same, as that constitutes the greatest part of the total manufacture cost. In addition, since this investigation was solely focused on the effect of coarse RMAc, the content of natural fine aggregates was maintained. Thus, water, cement, and fine aggregates content remained unaltered, whereas 50% of the total weight of the coarse natural aggregates was replaced by 4/20 mm recycled mixed ceramic aggregates that were pre-saturated before incorporation to the mix. A 50% replacement ratio was chosen based on the limit replacement values suggested in the literature review [11,14,17,39,40]. In addition, this RMAc content represents a significant substitution of natural aggregates, with the consequent saving of natural resources, and allows the creation of a concrete with similar characteristics to the control concrete. Regarding the need to take into account the greater water absorption of RMAc, the pre-saturation technique was preferred to mixing water compensation, since the latter could lead to bleeding risks that would alter the interfacial transition zone (ITZ) [41,42]. Nonetheless, in order to ensure correct pre-saturation practice, a detailed study regarding the absorption properties of the recycled aggregates must be performed on a case by case basis. In a previous investigation [36], it was found that, in order to achieve improvements in the consistency of the recycled concrete, the RMAc employed in this study requires a 3 min pre-saturation in potable water to reach 47.5% of the water absorbed at maximum saturation, which would require a 10 day immersion. Table 3 shows the detailed proportions of the different raw components used in the manufacture of the recycled concrete mixture (RC).

### 2.3. Non-Structural Precast Concrete Elements

Both curb units and paving blocks are produced with a single concrete throughout, and thus are considered monoblock non-structural precast elements [43]. The test concrete specimens were manufactured following the instructions outlined in References [44,45]. After casting, all test specimens were finished with a steel trowel, and were immediately covered with plastic film to avoid any water loss due to evaporation. After 24 h, all the specimens were demoulded and cured under water at 20 ± 2 °C.

**Figure 3:**
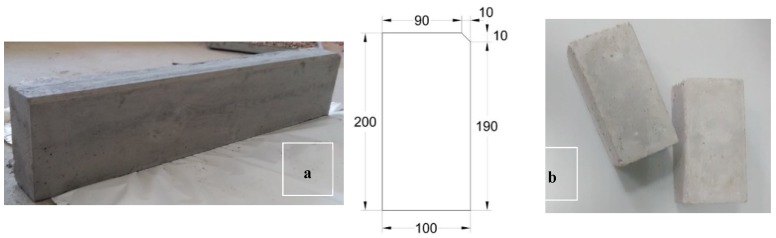
(**a**) General appearance and cross-sectional dimensions (mm) of the recycled curbstone. (**b**) General appearance of the recycled paving blocks.

The produced curbstones presented a 200 × 100 mm cross section and 1000 mm length, with an intended use as delineation of pedestrian walkways—Class A2, conforming to Reference [43]. Figure 3a illustrates both the general appearance and the cross-sectional dimensions of a curb unit for use in pedestrian sidewalks. The paving blocks were manufactured following the dimensions suggested by Prefabricados de Hormigón Pavimentos Páramo S.L. Hence, paving blocks of 200 mm length, 100 mm width and 80 mm height were produced (Figure 3b), since this typology is one of the most employed.

### 2.4. Tests

An experimental programme was carried out in order to evaluate the mechanical and microstructural properties of the recycled concrete made with a 50% substitution of coarse natural aggregates by RMAc. To compare the performance of the recycled concrete, commercially available conventional non-structural precast elements produced by Prefabricados de Hormigón Pavimentos Páramo S.L., i.e., analogous curb units and paving blocks to those produced in the laboratory, were employed.

#### 2.4.1. Consistency

The consistency, as an indirect measurement of workability, of the recycled concrete was determined by means of the Vebe test [46]. This test was performed on a sample obtained in accordance with Reference [47] immediately after the mixing stopped.

#### 2.4.2. Density

According to Reference [48] the hardened density of the recycled concrete, both in the saturated and oven-dried state, was determined as the average value from four prismatic specimens (200 × 100 × 80 mm) made with RMAc after 28 days of curing in water.

#### 2.4.3. Surface Finish and Dimensions

For both curb units and paving blocks, the requirements for visual aspects (appearance, texture, and colour) and dimensions were verified following the guidelines established in References [49,50,51] respectively.

#### 2.4.4. Compressive Strength

According to the standard in Reference [52], the characteristic compressive strength of the concrete was determined at 28 days for three cylindrical specimens (150 mm diameter and 300 mm height), meeting with the shape and size requirements of Reference [44], by means of a hydraulic press conforming to Reference [53]. The compressive test was preceded by capping of the trowelled surface to achieve a smooth surface for uniform distribution of the load during testing.

#### 2.4.5. Flexural Strength of Curbstones

The flexural strength of eight curb units was assessed conforming to References [49,50], and the Spanish national complement [43] to the aforementioned standard, on 28 days old specimens.

#### 2.4.6. Splitting Tensile Strength of Paving Blocks

According to Reference [51], the average splitting tensile strength of eight paving blocks was measured on 28 days old specimens.

#### 2.4.7. Microstructure

The microstructural studies, both SEM images and energy dispersive X-ray (EDX) elemental mappings, were conducted by a Hitachi S-4800 scanning electron microscope (Hitachi Group, Tokyo, Japan) with tungsten as X-ray source, a Si/Li detector and a Bruker XFlash 5030 EDS analyser (Bruker, Berlin, Germany). The standard preparation of the samples consisted of their placement in a metallic holder by means of a bi-adhesive graphite film and a subsequent carbon coating to ensure conductivity and avoid signal masking.

#### 2.4.8. Porosity

Mercury intrusion orosimetry (MIP) was used to determine the porosity and pore size distribution of 28-day cured concrete samples. The tests were developed using a Micromeritics AutoPore IV 9500 porosimeter (Micromeritics Corporate, Norcross, GA, USA), which operates in the pressure range 0.0034–227.5270 MPa over a pore diameter range of 0.006 µm to 175 μm. The samples were dried to constant weight at 40 °C and degassed with a vacuum pump for 30 min in order to ensure moisture removal.

#### 2.4.9. Water Absorption

Water absorption tests were carried out in four precast paving blocks according to Reference [51], and in three precast curbs according to References [49,50].

#### 2.4.10. Abrasion Test

The abrasion test was performed on three paving blocks and three curbstones, according to References [49,50,51] respectively.

#### 2.4.11. Freeze–Thaw Test

The freeze–thaw resistance in the presence of sodium chloride solution was evaluated in 28-day cured concrete based on References [49,50,51]. After a leakage test to guarantee correct sealing, a 5 mm thick layer of freezing medium (3 wt % NaCl) was placed on each specimen. Casting surfaces were tested. To avoid evaporation and concentration changes throughout the experiment, a plastic film was used to cover each sample. Subsequently, the specimens were introduced into a freezing chamber at the beginning of a freeze–thaw cycle, with temperatures ranging from −18 °C to 20 °C in 24 h. During the thawing phase of the 7th, 14th, 21th, and 28th cycles, each specimen was rinsed with tap water into a filter paper to collect the scaled material, which afterwards was dried at 105 °C over at least 24 h. Finally, the same quantity of freezing medium was poured again onto the test surface and the specimen was returned to the freezing chamber at the beginning of a new freeze–thaw cycle. Freeze–thaw resistance was assessed by subsequent weighing of surface mass loss per unit area.

#### 2.4.12. Electrical Resistivity

The electrical resistivity was tested according to Reference [54], at 20 ± 2 °C in 28-day saturated surface-dry specimens. Three 75 × 150 mm cylindrical specimens were used for each type of concrete.

## 3. Results and Discussion

### 3.1. Consistency and Density

A Vebe time of 9 s was obtained in the test of workability of both control concrete and recycled concrete, implying a dry consistency. According to several authors [10,55], these values are appropriate for precast concrete elements, and besides, no problems in workability were detected when placing, compacting, or casting the test specimens.

Regarding the hardened density of recycled concrete, the average value of prismatic specimens was 2295 and 2121 kg/m^3^ for the saturated and oven-dried states, respectively. Several studies have found that there is a density loss in recycled concretes when coarse natural aggregates are replaced by recycled mixed aggregates [56,57,58,59], due to the presence of adhered mortar, clay-based particles, and floating materials in the RMAc. These particles are responsible for the higher porosity of RMAc, and consequently, lower hardened density values of the recycled concrete. For curbstones made with a 50% substitution of recycled mixed aggregates, Reference [14] reported a density of 2240 kg/m^3^, which is similar to the results achieved in this research work and is lower than the standard density of a conventional concrete, 2300 kg/m^3^ [23].

### 3.2. Surface Finish and Dimensions

Both the dimensional requirements and the tolerance limitations established for curb units and paving blocks in their respective standards [49,50,51] were satisfied by the test specimens. Regarding the surface finish, the use of RMAc did not alter the fulfilment of the visual requirements. None of the specimens evaluated presented cracks or flaking. Moreover, the texture and colour were similar to the industrial specimens. Therefore, the curb units and paving blocks satisfied all these requirements.

### 3.3. Mechanical Properties

#### 3.3.1. Compressive Strength

**Figure 4:**
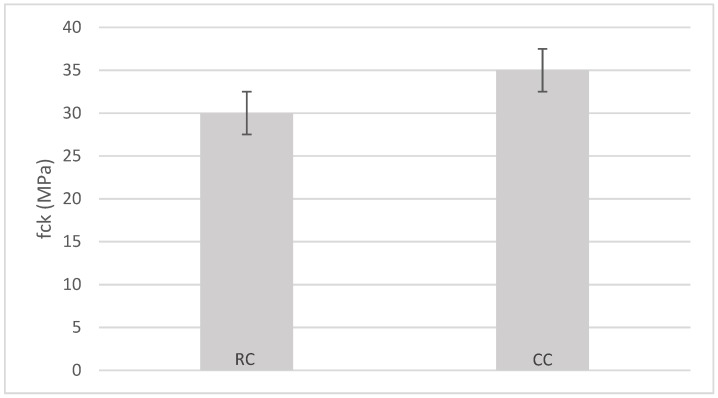
Characteristic compressive strength at 28 days (MPa) for recycled concrete (**RC**) and control concrete (**CC**).

According with Figure 4 the recycled concrete exhibited a characteristic compressive strength of around 30 MPa at 28 days of curing, whereas the reference concrete reached 35 MPa. Thus, a decrease of approximately 14% was observed in the compressive strength of the recycled concrete. The presence of adhered mortar, and ceramic materials, as well as some other impurities like wood, plastic, gypsum, etc., in the RMAc may have been responsible for the compressive strength loss [4]. A wide range of 28-day compressive strength declines have been reported by numerous authors in studies where a replacement of about 50% of natural coarse aggregate by CDW was carried out. For instance, a 26.8% decrease in compressive strength was observed in Reference [59], and a 10% loss was reported in Reference [60]. Nonetheless, others have considered that replacements up to 60% do not significantly affect the mechanical properties of recycled concrete, as stated in Reference [16].

#### 3.3.2. Flexural Strength of Curbstones

**Figure 5:**
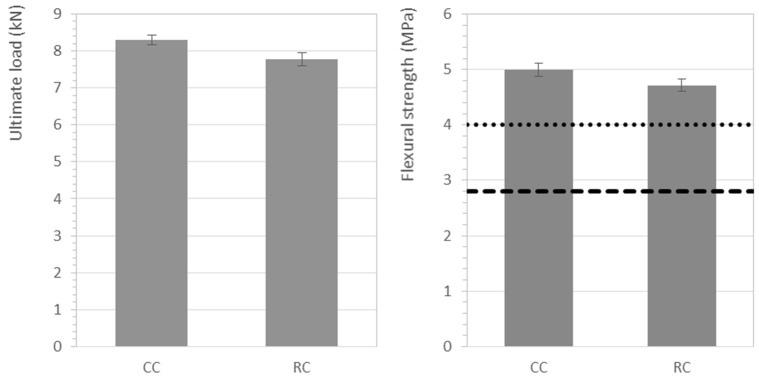
Mechanical characterization of the conventional (CC) and recycled (RC) curb units, where the dashed and dotted lines represent the Class 1 and 2 thresholds respectively.

Eight curb units of the conventional and recycled concretes were subjected to flexural tests. The results for ultimate load and flexural strength are shown in Figure 5. For the recycled specimens, all individual results of failure load were over 4.65 kN, with an average of 7.7 kN. Regarding the flexural strength, the individual values of the 8-set sample exceeded 4 MPa, while the average was 4.71 MPa. Therefore, according to Reference [43], the recycled concrete curb units must be considered class 1 and will exhibit an S marking. This marking means that they can be used in pedestrian zones or areas with light traffic.

Nonetheless, the use of a 50% substitution of the coarse natural aggregate by RMAc resulted in a slightly poorer performance of the curbstones, around 5%, when compared to those made with conventional concrete. Similar mechanical performance and strength reductions have been found by other authors [14,39,61]. On the contrary, Reference [11] only observed decreases in the flexural strength for replacements greater than 70%, and a 33.6% reduction in the flexural strength was reported for a complete substitution.

#### 3.3.3. Splitting Tensile Strength of Paving Blocks

**Figure 6:**
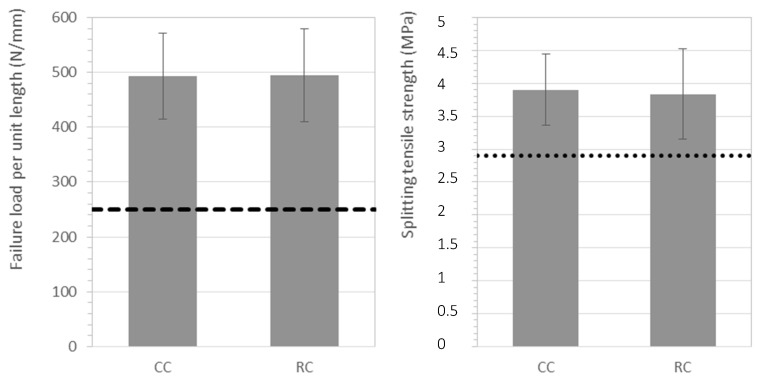
Mechanical characterization of the conventional (CC) and recycled (RC) paving blocks. The dashed and dotted lines represent the failure load and the splitting tensile strength thresholds.

Eight paving blocks of the conventional and recycled concretes were subjected to splitting tensile tests. The results of failure load per unit length and the splitting tensile strength are shown in Figure 6. For the recycled concrete, all paving block specimens exhibited a failure load per unit length above the minimum of 250 N/mm established in the standard EN-1338 referenced [51], while the average failure load per length reached nearly 500 N/mm. Regarding splitting tensile strength, all samples exceeded the minimum established value of 2.9 MPa for individual specimens. In addition, recycled paving blocks presented an average splitting tensile strength of 3.9 MPa, above the 3.6 MPa threshold established in the standard.

Thus, the tensile strength comparison of the conventional and recycled concretes showed that a 50% replacement ratio of the natural coarse aggregates by RMAc did not affect the mechanical performance of the paving blocks. Similarly, Reference [16] reported that up to a 50% replacement of coarse masonry-derived recycled aggregates also resulted in recycled concrete exceeding the 3.6 MPa tensile splitting strength threshold. Moreover, their study concluded that replacements up to 60% still met the requirements established for paving blocks. The study presented in Reference [40] observed gains in the transverse strength of paving blocks made with a 50% replacement of both fine and coarse natural aggregates by recycled concrete aggregates. However, Reference [12] noticed that increasing the percentages of brick materials in recycled aggregates decreased the splitting tensile strength. But later on, Reference [13] also reported that incorporating a small amount, about 10%, of crushed tiles and bricks into the total recycled aggregates resulted in a greater tensile splitting strength of paving blocks. The authors attributed those results to an improved ITZ due to high water absorption of ceramic materials, which made possible a better penetration of the cement paste, and to an increased amount of finer particles that filled the voids and reduced the porosity. However, for a complete substitution of coarse natural aggregates by recycled crushed brick aggregates, Reference [10] reported that the average splitting tensile strength did not fulfil the strength limit established.

#### 3.3.4. Microstructure

**Figure 7:**
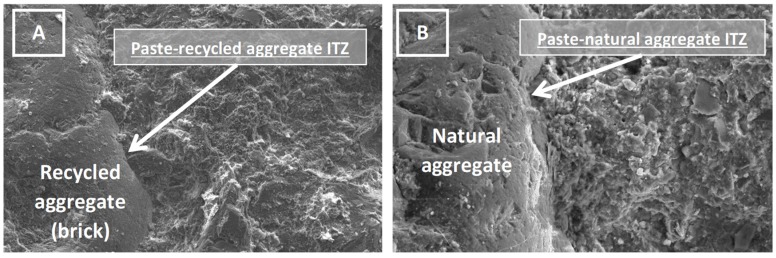
(**A**) Scanning electron microscope image of recycled concrete. (**B**) Scanning electron microscope image of conventional concrete.

The SEM images of the concrete samples display the paste-aggregate ITZ, showing the interface between the paste and a recycled brick aggregate (Figure 7A), and between the paste and a natural aggregate (Figure 7B). In both cases, it can be observed that the cement paste was able to cover the aggregates surface appropriately. No negative effects were detected due to the presence of recycled aggregates, indeed, the ITZ around the recycled ceramic aggregates (Figure 7A) seemed similar or even better than between paste and natural aggregates (Figure 7B).

Some authors relate the higher water absorption [6,7,8] displayed by recycled aggregates to a weaker ITZ, due to a worse cement hydration around the aggregate. For instance, References [41,62], who made recycled concrete by replacing natural aggregates by recycled aggregates without any water compensation, claimed that the ITZ between recycled aggregates and cement paste was weaker than the bond developed between the paste and the natural aggregates. However, in this investigation, the pre-saturation [36] of RMAc before their addition to the concrete mix was employed as a means to solve the water absorption drawback commonly reported when employing recycled aggregates. In addition to the beneficial effect of the pre-saturation observed in the consistency development of the recycled concrete, this technique was also able to improve the ITZ between recycled aggregates and cement paste, since the surfaces of aggregates after soaking are in a more receptive state to be joined with the cement powder. The work of Reference [63], using a similar technique, where recycled aggregates were mixed with water before being added to the cement in the mixing process, achieved a stronger and denser interfacial zone in the recycled concrete compared to the ITZ in recycled concrete made with a normal mixing approach (recycled aggregates in dry state).

On the other hand, the pre-saturation technique is also known for causing a certain decline in the mechanical properties of recycled concrete [36,41,64,65], which was also observed in this study.

**Figure 8:**
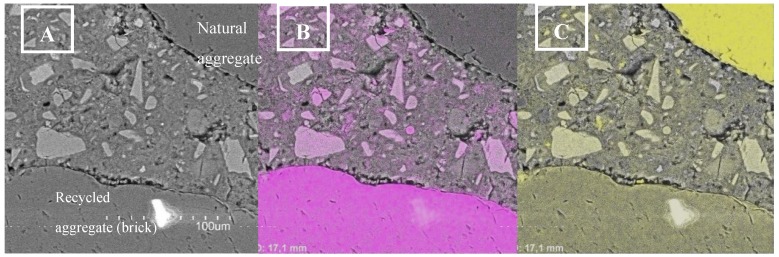
Elemental maps of recycled concrete: (**A**) Base image. (**B**) Elemental mapping for aluminium (magenta). (**C**) Elemental mapping for silicon (yellow).

The microstructure study also entailed an analysis of the elemental mapping of a 28-day recycled concrete sample obtained by SEM (Figure 8). In the figure, where the aluminum is represented by the magenta color and the silicon is signified by the yellow color, it is possible to observe that both kinds of aggregates developed similarly adequate ITZs. Furthermore, a slight improvement can be distinguished in the ITZ between the paste and the recycled aggregate by the more homogeneous distribution and strong vividness of the magenta color around the brick particle, with fewer pores observed around the perimeter of recycled aggregate in comparison with those ones around the natural aggregate. This result may be attributed to the pre-saturation of the recycled aggregates prior to the mixing stage, since the natural aggregates used in the recycled concrete (50% non-replacement) were added to the batch without any soaking pre-treatment.

**Figure 9:**
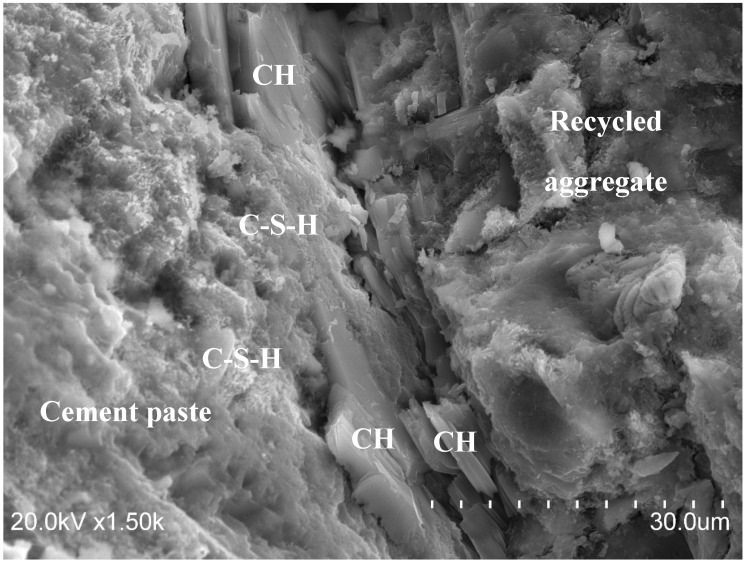
SEM image of recycled concrete, showing the cement hydration products.

Figure 9 illustrates the microstructure of the recycled concrete, displaying the cement paste on the left side and the recycled aggregate on the right side of the image obtained by SEM. In this figure, portlandite crystals can easily be detected in the interfacial transition zone between the cement paste and recycled aggregate. These hexagonal crystals, with a size higher than 10 µm in most cases, are similar to those reported for different types of concretes [66,67,68,69,70], including some recycled aggregates. Additionally, some calcium silicate hydrate gels (C–S–H) were also observed close to the portlandite crystals. These two solid phases are the principal cement hydration products in a conventional concrete [71,72,73], so it is possible to establish that the behavior of cement hydration in recycled concrete is not affected, due to replacement of natural aggregates by recycled ones.

Another point worth mentioning in Figure 9 is the lack of ettringite detected; their typical needle shape was not observed. Therefore, the problem considered by authors such as in Reference [38], who claimed that high contents of gypsum could generate expansion problems in recycled concrete owing to the delayed formation of ettringite, is not expected to occur in the recycled mixture tested in this paper.

### 3.4. Durability Properties

#### 3.4.1. Porosity

Despite presenting certain limitations, such as the necessity of assuming a pore shape model, the so-called ink-bottle effect, and the possible changes in microstructure resulting from de pre-condition of the sample, porosity is generally accepted and used as durability indicator for hardened concrete [74,75].

**Figure 10:**
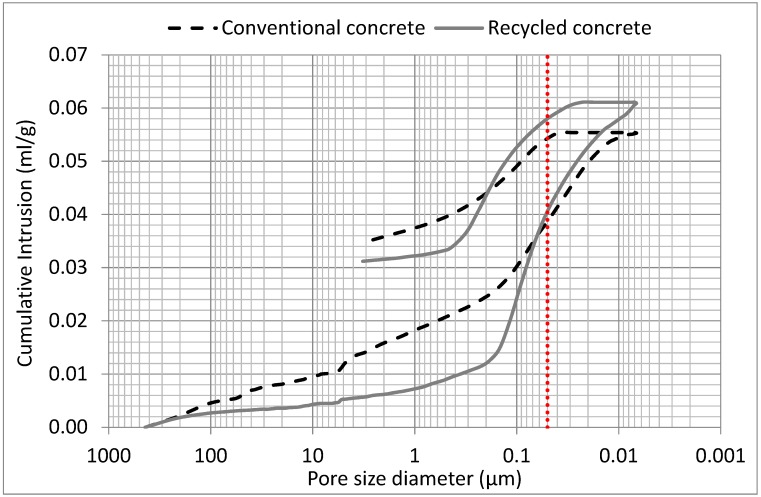
Pore size distribution of conventional and recycled concrete sample at 28 days.

The relationship between the cumulative intrusion and pore size diameter of the recycled and conventional concrete mixtures (i.e., recycled aggregates + new cement paste and natural aggregates + new cement paste), obtained by mercury intrusion porosimetry (MIP) at 28 days, is shown in Figure 10. As can be observed in the intrusion plots, the cumulative intrusion of mercury into the recycled concrete within the 0.06 µm to 439 µm (maximum pore diameter detected) pore size diameter range was significantly lower than that of the conventional concrete. The same occurred with the extrusion plot for pore size diameters higher than 0.2 µm. Therefore, these results suggest that recycled concrete made using RMAc could result in a suitable durability performance, and that it could be even more durable than conventional concrete, since fewer pores with higher pore size diameter have been linked to improved concrete durability [74,76]

According to Reference [77], who tested the porosity of concrete made with four different recycled concrete aggregates, the volume of pores between 0.05 µm and 2 µm tended to increase with the presence of attached mortar. In view of the results shown in Figure 10, it could be stated that the presence of ceramic fraction positively affected the pore size distribution of recycled concretes, since the growth trend in the pore volume was delayed until the 0.2–0.4 µm pore size mark. In this context, it is worth mentioning that former researchers [74,76] have stated that the number of pores with a size lower than 0.1 µm exerts a minor role in concrete durability.

Additionally, the effect of the pre-saturation technique should be considered in the porosity results obtained. Reference [78], who analyzed the effect of aggregate saturation level (0%, 50%, and 100%) on the properties of the resulting concrete, stated that the cumulative porosity, and the volume of pores with diameters between 0.1 µm and 0.4 µm (mesoporosity), depended on the initial degree of saturation of the aggregates. Their findings concluded that a 50% saturation level improved mechanical strength and decreased mesopore volume. Therefore, those results support use of the pre-saturation technique as followed in this research, which brought the RMAc to a saturation level around 47.5% [36], as a means to control the mesoporosity and achieve a suitable performance in recycled concrete.

Concerning the total porosity at 28 days, the value registered for the conventional concrete was slightly lower (12.37%) than that of the recycled concrete (12.44%), since the volume of pores with diameter lower than 0.06 µm in the recycled concrete was higher. The work of References [79,80], who tested analogous concretes (i.e., presenting w/c ratios ranging from 0.5 to 0.55), obtained similar results for recycled concrete porosity. Nevertheless, other research works in which similar recycled aggregates were employed in the concrete mixes have reported greater values of total porosity in the recycled mixtures. Reference [81] observed values fluctuating between 16.9% and 21.5% for recycled aggregate concrete with a w/c ratio of 0.5. In the work of Reference [82], recycled concrete manufactured with 30% and 100% recycled concrete aggregate replacements displayed porosity values of 13% and 16%, respectively. Reference [83], who employed 15%, 20%, and 25% recycled sanitary ceramic aggregate in the manufacture of concrete, obtained recycled concretes with porosity values of 15.98%, 16.21%, and 16.38%, respectively.

Consequently, taking into account all of the above, it can be stated that the pore network model exhibited by the recycled concrete in this study had positive effects on the mechanical properties and durability of the manufactured concrete, some of which are studied in detail in the present paper.

#### 3.4.2. Water Absorption

The precast recycled elements (paving blocks and curbstones) presented an average water absorption of 8%, greater than the 6% limit required to satisfy requirements for Class 2 weather resistance. As a result, these precast recycled concrete elements must be classified into Class 1. On the other hand, the precast paving blocks and curbs manufactured with the control concrete exhibited a water absorption of 4%, which made it possible to classify them as Class 2 and B marking (average water absorption ≤ 6%). Therefore, it was ascertained that the presence of RMAc exerted a strong influence on the increase of water absorption of the hardened concrete.

The study of Reference [10], who tested paving blocks and flags made with crushed brick as a coarse aggregate, concluded that a replacement of 60% induced a water absorption value of 13.1%. For a replacement of 20% of the fine aggregate and 30% of the coarse aggregate by crushed brick, the water absorption value was lower at 11.7%. However, some authors [16,17] have provided more encouraging findings, since they established that the water absorption requirement (<6%) could be met with a maximum 55% replacement level of natural coarse aggregate by masonry-derived aggregates. Later on, Reference [84] reduced the permissible replacement level to 40%, in order that a recycled concrete incorporating recycled mixed aggregates could comply with the maximum water absorption requirement of 6%.

High values of water absorption are commonly observed in concrete mixes containing recycled mixed aggregates [85,86]. However, some studies have concluded that concretes made with recycled aggregates of a ceramic nature exhibit a superior durability performance, due to the specific capillary water absorption presented by that type of recycled concrete. Reference [87], who manufactured recycled concrete curbs and floor blocks, noticed that masonry wastes and unbounded aggregates were not as absorbent as concrete wastes. Reference [15] showed that the water absorption of concrete blocks containing 100% fine crushed ceramic was 34.9% lower than that of control blocks, whereas an increase of 46.2% in the water absorption of recycled concrete blocks was observed when a 100% coarse crushed ceramic replacement was employed.

#### 3.4.3. Abrasion Test

Abrasion resistance is one of the most common parameters used to measure the durability of precast concrete. According to Reference [88], abrasion resistance is governed by both the properties of the aggregates employed and the compressive strength of the specimen. The abrasion wear value displayed by the recycled paving block and curbstone samples tested in this study was 19 mm. In both cases, the abrasion wear was within the tolerance limitations to be classified as Class 4 and I marking (≤20 mm abrasion wear). As a matter of fact, the recycled mixtures exhibited an improved performance compared to the control precast elements, which only achieved Class 3 and H marking, since their abrasion wear average was 23 mm. Therefore, the precast pieces supplemented with RMAc could be used in areas of very heavy pedestrian and vehicular traffic. The improvement of the abrasion resistance can be attributed to the denser paste and stronger bond at the interface between cement paste and the aggregates displayed by the recycled concrete.

For replacements up to 75%, Reference [14] reported that the abrasion resistance of recycled paving blocks and curbstones was comparable to that of conventional precast elements, whereas decreases were observed for 100% substitutions. For a complete replacement of both natural fine and coarse aggregates, Reference [13] reported that the presence of tiles, bricks, glass, or wood did not hinder the fulfilment of the maximum 23 mm abrasion resistance requirement of Class 3. Reference [12] obtained similar results for recycled concretes incorporating up to 75% of brick materials. Reference [89], who investigated the effect of crushed ceramic in the production of interlocking paving units, concluded that samples with 50% replacement of natural coarse aggregate by ceramic satisfied the requirement of Class 3.

On a similar note, Reference [22] claimed that the size of the abrasion tracks in a concrete mixture with 20% RMA remained practically the same in comparison with the reference sample, meanwhile the abrasion wear increased slightly, and thus a lower abrasion resistance was observed, when a 40% substitution was carried out. Both References [90,91], who noticed that the inclusion of ceramic aggregates resulted in improved abrasion behavior of recycled concrete, attributed the improved resistance to the harder surface exhibited by the ceramic materials compared to the natural aggregates, despite being the former more fragile. However, Reference [10] argued that the use of increasing contents of recycled ceramic aggregate decreases the resistance to abrasion compared to reference concrete. Nonetheless, the authors concluded that all paving blocks and flags made with crushed brick as recycled aggregate in their study were able to satisfy the minimum requirements of the European standards.

#### 3.4.4. Freeze–Thaw Test

**Figure 11:**
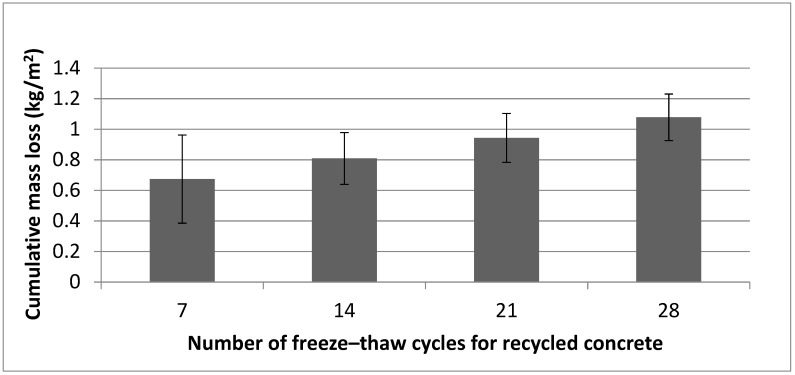
Cumulative mass loss of recycled aggregate concrete samples versus freeze–thaw cycles.

Figure 11 shows the evolution of the cumulative mass loss suffered by the recycled concrete samples across 28 cycles of freeze–thaw. As can be seen, the amount of detached material tended to decrease as time passed. In early cycles, the amount of detached material was significant, and consisted mainly of cement paste from the surface of the concrete samples. After the detachment of the first layer, lower rates of mass loss were observed, as the presence of aggregates increased the resistance of concrete to frost-salt scaling. In comparison with cement paste, the aggregates exhibited a porous network that greatly hindered the water flow, improving their resistance to freeze–thaw cycles. Furthermore, the improvement can also be attributed to the recycled aggregates, which allowed the existing water in the cement paste to move through them more easily than through the natural aggregates. In this case, recycled aggregates acted as an air entraining agent and provided additional spaces to decrease the hydraulic pressure developed during the ice formation [92,93,94,95].

The average total mass loss after the 28 cycles of freeze–thaw was found to be 1.0 kg/m^2^, which was within the Class 3 tolerance limit commonly established by the standards of precast concrete production, such as those concerning [49,51,96]. Moreover, the individual mass loss requirement, which has to be less than 1.5 kg/m^2^, was also reached, since the maximum loss value obtained was 1.3 kg/m^2^.

The control concrete exhibited a similar value for this test, the average data of individual mass loss was 0.9 kg/m^2^ and the individual data also was less than 1.5 kg/m^2^ with a limit value of 1.3 kg/m^2^.

Several authors [97,98] who tested recycled aggregate concrete against freeze–thaw weathering conditions, have stated that the frost resistance of this kind of concrete is not satisfying under severe climate conditions. Therefore, some authors have attempted some modifications to the proportioning of recycled concrete as a means to improve the freeze–thaw resistance. For instance, [99] stated that a minimum cement content of 395 kg/m^3^ was necessary to satisfy the ASTM specifications for the freezing and thawing resistance of paving blocks, even for those made with conventional concrete. Reference [100] reported that the use of chemical admixtures, particularly air entrainment agents, could palliate the negative effect of recycled aggregates on the durability of concrete and allow to produce mixtures as durable as the natural aggregate concrete. Reference [97] also stated that recycled aggregate concrete with a 5% entrained air content was found to be as durable as conventional concrete since entrained air voids act as stress relief sites that lead to enhanced freeze–thaw durability [101]. Finally, Reference [102] pursued the manufacture of recycled two-state concrete (TSC), which is distinguished by its high coarse aggregate content and the direct placement of the aggregates in the mold prior to be injected with a special grout, as a solution to the problem. However, their findings pointed out that TSC mixtures made with recycled concrete aggregates were not suitable for pavement construction in cold climates characterized by frequent freezing-thawing cycles.

On the contrary, Reference [10], who used crushed bricks in the production of concrete precast elements for pedestrian use, stated that the mass loss for all type of concrete blocks and flags was less than or equal to 1.0 kg/m^2^ after the freeze–thaw cycles, and thus satisfied the requirements for the best weather resistance class according the European standards. Furthermore, Reference [103], who evaluated the recycled concrete aggregates for their suitability in construction activities, proved the ability of aggregates to withstand the disintegration due to freeze–thaw weathering cycles through the soundness test. Therefore, the authors endorsed the potential use of recycled concrete in both road construction and in concrete structures. Moreover, recycled concrete can also be used in concrete elements that have no restrictions placed on its use, such as special roof elements, box culverts, etc. In addition, it should be taken into account that freezing and thawing usually do not occur in tropical and subtropical areas. Then, freeze–thaw resistance is not necessarily a generally limiting factor for recycled concrete [104].

#### 3.4.5. Electrical Resistivity

One of the main parameters that affects the electrical resistance of the concrete is the properties of the coarse aggregate. Reference [105] studied the effects of coarse aggregates on the electrical resistivity of Portland cement concrete, and stated that concrete resistivity is sensitive to the inclusion of coarse aggregates. According to this study, the coarse aggregates serve as a direct electrical obstacle; therefore, they increased the electrical resistivity. The authors, who also tested the variation in coarse aggregate size and type, observed that those aggregate properties exerted no effects on the electrical resistivity. The study presented by Reference [6] shows that electrical resistivity decreased in recycled concrete with an increase of recycled concrete aggregates.

**Figure 12:**
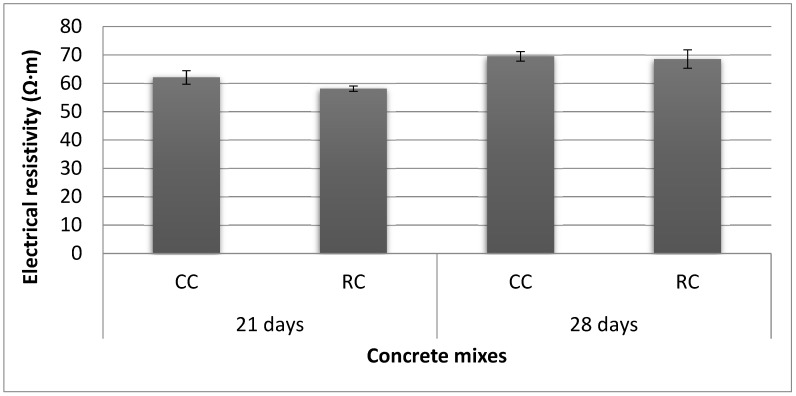
Electrical resistivity of the samples, control concrete (CC) and recycled concrete (RC).

Figure 12 displays the evolution of the electrical resistivity of conventional and recycled samples. In the first stage, the electrical resistivity exhibited by the conventional concrete samples was higher (6.4%) than that of the recycled concrete samples after 21 days curing. However, when both concrete mixes reached 28 days of curing, the electrical resistivity of the recycled concrete samples was almost equal to that of the conventional concrete samples (i.e., showing a slight decrease of 1.4%). Over time, the electrical resistivity values showed a general tendency to increase in both cases, due to the porosity decrease related to the curing age. Nevertheless, this pore network evolution was more powerful in the recycled concrete [74], which is considered a factor in the improvement of the durability behavior of concrete incorporating recycled coarse aggregate. These results are consistent with those encountered in other studies [106,107], where it has been stated that the use of recycled concrete aggregates reduced the electrical resistivity.

Conversely, Reference [108], who manufactured recycled concrete with a 20 and 25% replacement of natural coarse aggregate by recycled ceramic sanitary ware aggregate, reported that the electrical resistivity values were 17% and 25% higher in the recycled concrete compared to conventional concrete, respectively. The authors attributed this effect to the insulating power of this type of industrial waste. Reference [109], who substituted the natural aggregate with recycled aggregates from porcelain electrical insulators, reported a lower corrosion potential in recycled samples. Reference [110] stated that the electrical resistivity of ceramic aggregates is in the order of 10^13^ Ω∙m, compared to 3.8 × 10^4^–1.2 × 10^12^ Ω∙m in natural gravel. In this regard, the electrical resistivity achieved by the recycled sample in the present research study at 28 days (43.15 Ω∙m) was similar to value obtained by Reference [108] for recycled concrete with a 20% aggregate replacement ratio, which bears witness to the insulating properties of the RMAc.

Furthermore, according to some research, the presence of ceramic in cement-based materials plays a positive role in the electrical resistance. Reference [111], who tested paste and mortar samples using Portland cement as cementitious material, and replacing it with different proportions of tile ceramic waste, claimed that mixtures with greater proportions of ceramic materials exhibited a rapid reduction in their conductivity, which indicates that the calcium and hydroxide ions (Ca^2+^ and OH^−^) of the dissolved lime were consumed by ceramic waste to generate cementitious products that precipitated.

## 4. Conclusions

From the results obtained in this manuscript, it is possible to conclude the following:

1. The use of recycled concrete, made with up to a 50% substitution of the natural coarse aggregates by RMAc aggregates, does not affect the general behavior of the concrete. In particular, it is worth mentioning the durability performance reached by the recycled concrete, which was able to comply with the requirements established in the standards for curbstones and paving blocks.
2. The effects of a 50% RMAc incorporation on the workability and density of concrete were acceptable, despite resulting in drier consistencies and lower densities.
3. The recycled concrete presented a good superficial finish, and a texture and colour comparable to the industrially produced specimens.
4. The mechanical resistances were the properties most affected by the partial substitution. Although samples complied with the requirements set forth in the standards, decreases in the compressive and flexural strength were observed. Nevertheless, the splitting tensile strength displayed by the recycled paving blocks was barely affected by the substitution, and showed similar values to those obtained by the conventional concrete.
5. The SEM, backscattered-electron (BSE) and MIP analysis revealed that a 3 min RMAc pre-saturation effectively palliated their greater water absorption, and thus, a good cement hydration was achieved within the recycled paste. Moreover, an adequate covering effect of the cement paste around the ceramic recycled aggregates was observed, and the thickness of the ITZ between recycled aggregates and cement paste indicated an improved bond compared to the interface developed around the natural aggregates.
6. The indirect measure of compactness, evaluated by the electrical resistivity of the samples, revealed that both concretes presented a similar behavior.
7. The results regarding the durability of the recycled concrete, which was evaluated through different tests: water absorption, abrasion, and a freeze–thaw test, showed that the major effect was that noticed for water absorption, which surpassed the limit established in the standards (6%) and reached values of 8% and 9% for blocks and curbstones, respectively. Despite this drawback, it was possible to ascertain that the abrasion and freeze–thaw resistance of the conventional and recycled concrete was similar, even slightly better in the recycled concrete.

Despite some slightly unfavorable results, these new mass concrete paving elements appear very strong, and show the potential for real applications of these secondary aggregates for the synthesis of concrete. More future research is still necessary in order to provide useful tools for future massive use of these materials. Among others, our research group is developing a recycled concrete using not only a partial substitution of aggregates, but a complete substitution of commercial Portland cement for recycled cement, using a mixture of CEM I and ceramic components obtained from CDW. The potential of both coarse and fines aggregates should be also studied, modifying the percentage of substitution in an attempt to decrease the use of natural raw materials and contribute to a circular economy.

## Figures and Tables

**Table 1:** Physical and mechanical properties of RMAc.

Properties	Test Result	Limit Value	Standard Applied
Maximum particle size (mm)	20	-	EHE-08 (2008); EN 933-1 (2012)
Minimum particle size (mm)	4	4	EHE-08 (2008); EN 933-1 (2012)
D/d ratio	5.0	≥1.4	EHE-08 (2008); EN 933-1 (2012)
Granulometric modulus	7.67	-	UNE 7295 (1976)
Content of particles < 4 mm (%)	5.03	5	EHE-08 (2008)
Undersize particle content (%) Sieve d	5.03	≤10 (EHE-08) <10 (UNE 146121)	EHE-08 (2008); UNE 146121 (2000); EN 933-1 (2012)
Oversize particle content (%) Sieve 2D	0	0	EHE-08 (2008); UNE 146121 (2000); EN 933-1 (2012)
Oversize particle content (%) Sieve D	2.21	<10	EHE-08 (2008); UNE 146121 (2000); EN 933-1 (2012)
Fines content (%)	0.04	≤1.5 (EHE-08) ≤1 (UNE 146121)	EHE-08 (2008); UNE 146121 (2000); EN 933-1 (2012)
Sand equivalent (SE4 or SE)	38.10	>70–75 ^(1)^	EHE-08 (2008); EN 933-8 (2012)
Apparent density (Mg/m^3^)	2.53	-	EN 1097-6 (2014)
After oven-drying density (Mg/m^3^)	2.08	-	EN 1097-6 (2014)
Saturate surface density (Mg/m^3^)	2.26	-	EN 1097-6 (2014)
Water absorption (%)	8.53	≤7	EHE-08 (2008); EN 1097-6 (2014)
Flakiness index (%)	14.75	≤35	EHE-08 (2008); EN 933-3 (2012)
Los Angeles coefficient (%)	40.99	≤40–50 ^(2)^	EHE-08 (2008); EN 1097-2 (2010)

Notes: (1) The Spanish standard for concrete (EHE-08) allows the use of aggregates with sand equivalent minor to 70 for construction works in environments type X0, XC1, XC2, XC3, and XC4. The value increases up to 75 for all other cases. (2) It is possible to produce mass or reinforced concrete with a characteristic compressive strength fck ≤ 30 MPa using coarse aggregates with LA coefficients between 40 and 50, if specific studies endorse their use without detriment to concrete performance.

**Table 2:** Non-floating components of the recycled aggregates.

Components of Recycled Mixed Ceramic Aggregates (RMAc)	Percentage (wt %)
Unbound aggregates (natural aggregates without cement mortar attached)	44.11
Ceramics (bricks, tiles, stoneware and sanitary ware…)	33.56
Concrete and mortar (natural aggregates with cement mortar attached)	17.51
Asphalt	0.44
Glass	0.75
Gypsum	3.47
Other impurities (wood, paper, metals, plastic…)	0.16
Total	100

**Table 3:** Mix proportions per cubic metre of recycled concrete (RC) and control concrete (CC).

Dosification Per Cubic Meter	RC	CC
Total effective w/c ratio (-)	0.50	0.50
Water (L)	155.21	155.21
Cement (kg)	312.50	312.50
Sand 0/4 mm (kg)	96.98	96.98
Sand 0/5 mm (kg)	441.81	441.81
Gravel 4/10 mm (kg)	242.46	484.92
Gravel 6/12 mm (kg)	80.82	161.64
Recycled mixed ceramic aggregate 4/20 mm (kg)	323.28	-

## References

[1] Ecoembes Presentación de Resultados 2017. https://www.ecoembes.com/sites/default/files/reciclaje-en-datos-2017.pdf.

[2] Eurostat (2016). Waste Statistics—Statistics Explained. http://ec.europa.eu/eurostat/statistics-explained/index.php/Waste_statistics.

[3] Spanish Ministry of Environment (2001). Resolución de 14 de Junio de 2001, de la Secretaría General de Medio Ambiente, por la que se Dispone la Publicación del Acuerdo de Consejo de Ministros, de 1 de Junio de 2001, por el que se Aprueba el Plan Nacional de Residuos de Construcción y Demolición [WWW Document]. http://noticias.juridicas.com/base_datos/Admin/res140601-1-mma.html.

[4] Bravo M., de Brito J., Evangelista L., Pacheco J. (2017). Superplasticizer’s efficiency on the mechanical properties of recycled aggregates concrete: Influence of recycled aggregates composition and incorporation ratio. Constr. Build. Mater..

[5] Bravo M., de Brito J., Pontes J., Evangelista L. (2017). Shrinkage and creep performance of concrete with recycled aggregates from CDW plants. Mag. Concr. Res..

[6] Kurda R., de Brito J., Silvestre J.D. (2019). Water absorption and electrical resistivity of concrete with recycled concrete aggregates and fly ash. Cem. Concr. Compos..

[7] Kurda R., Silvestre J.D., de Brito J. (2018). Life cycle assessment of concrete made with high volume of recycled concrete aggregates and fly ash. Resour. Conserv. Recycl..

[8] Pedro D., de Brito J., Evangelista L. (2018). Durability performance of high-performance concrete made with recycled aggregates, fly ash and densified silica fume. Cem. Concr. Compos..

[9] Carro-López D., González-Fonteboa B., Martínez-Abella F., González-Taboada I., de Brito J., Varela-Puga F. (2018). Proportioning, fresh-state properties and rheology of self-compacting concrete with fine recycled aggregates. Hormig. Acero.

[10] Jankovic K., Nikolic D., Bojovic D. (2012). Concrete paving blocks and flags made with crushed brick as aggregate. Constr. Build. Mater..

[11] López Gayarre F., López-Colina C., Serrano M.A., López-Martínez A. (2013). Manufacture of Concrete Kerbs and Floor Blocks with Recycled Aggregate from C&DW. Constr. Build. Mater..

[12] Poon C.S., Chan D. (2006). Paving blocks made with recycled concrete aggregate and crushed clay brick. Constr. Build. Mater..

[13] Poon C.S., Chan D. (2007). Effects of contaminants on the properties of concrete paving blocks prepared with recycled concrete aggregates. Constr. Build. Mater..

[14] Rodríguez C., Parra C., Casado G., Miñano I., Albaladejo F., Benito F., Sánchez I. (2016). The incorporation of construction and demolition wastes as recycled mixed aggregates in non-structural concrete precast pieces. J. Clean. Prod..

[15] Sadek W., El-Sayed A., Heniegal A.M. (2013). Production of solid cemente bricks using some types of solid wastes. Eng. Res. J..

[16] Soutsos M.N., Tang K., Millard S.G. (2011). Use of recycled demolition aggregate in precast products, phase II: Concrete paving blocks. Constr. Build. Mater..

[17] Soutsos M.N., Tang K., Millard S.G. (2011). Concrete building blocks made with recycled demolition aggregate. Constr. Build. Mater..

[18] Juan-Valdés A., Rodríguez-Robles D., García-González J., Guerra-Romero M.I., Morán-del Pozo J.M. (2018). Mechanical and microstructural characterization of non-structural precast concrete made with recycled mixed ceramic aggregates from construction and demolition wastes. J. Clean. Prod..

[19] Anon (2012). Guía Española de Áridos Reciclados Procedentes de Residuos de Construcción y Demolición.

[20] RC-16 (2016). Royal Decree 256, Cement Reception Instruction (RC-16).

[21] EN 197-1 (2011). Cement Part 1: Composition, Specifications and Conformity Criteria for Common Cements.

[22] Mas B., Cladera A., Bestard J., Muntaner D., López C.E., Piña S., Prades J. (2012). Concrete with mixed recycled aggregates: Influence of the type of cement. Constr. Build. Mater..

[23] EHE-08 (2008). Code on Structural Concrete (EHE-08).

[24] EN 12620+A1 (2008). Aggregates for Concrete.

[25] EN 933-1 (2012). Tests for Geometrical Properties of Aggregates—Part 1: Determination of Particle Size Distribution—Sieving Method.

[26] EN 933-3 (2012). Tests for Geometrical Properties of Aggregates—Part 3: Determination of Particle Shape—Flakiness Index.

[27] EN 1097-2 (2010). Tests for Mechanical and Physical Properties of Aggregates—Part 2: Methods for the Determination of Resistance to Fragmentation.

[28] EN 1097-6 (2013). Tests for Mechanical and Physical Properties of Aggregates—Part 6: Determination of Particle Density and Water Absorption.

[29] Abbas A., Fathifazl G., Burkan Isgor O., Razaqpur A.G., Fournier B., Foo S. (2008). Proposed Method for Determining the Residual Mortar Content of Recycled Concrete Aggregates. J. ASTM Int..

[30] Shi C., Li Y., Zhang J., Li W., Chong L., Xie Z. (2016). Performance enhancement of recycled concrete aggregate—A review. J. Clean. Prod..

[31] Tam V.W.Y., Tam C.M., Le K.N. (2007). Removal of cement mortar remains from recycled aggregate using pre-soaking approaches. Resour. Conserv. Recycl..

[32] Zhang J., Shi C., Li Y., Pan X., Poon C.-S., Xie Z. (2015). Influence of carbonated recycled concrete aggregate on properties of cement mortar. Constr. Build. Mater..

[33] Zhang J., Shi C., Li Y., Pan X., Poon C.-S., Xie Z. (2015). Performance Enhancement of Recycled Concrete Aggregates through Carbonation. J. Mater. Civ. Eng..

[34] Poon C.S., Chan D. (2006). Feasible use of recycled concrete aggregates and crushed clay brick as unbound road sub-base. Constr. Build. Mater..

[35] Yang J., Du Q., Bao Y. (2011). Concrete with recycled concrete aggregate and crushed clay bricks. Constr. Build. Mater..

[36] García-González J., Rodríguez-Robles D., Juan-Valdés A., Morán-del Pozo J., Guerra-Romero M.I. (2014). Pre-Saturation Technique of the Recycled Aggregates: Solution to the Water Absorption Drawback in the Recycled Concrete Manufacture. Materials.

[37] EN 933-11 (2009). Tests for Geometrical Properties of Aggregates—Part 11: Classification Test for the Constituents of Coarse Recycled Aggregate.

[38] Neville A.M. (1995). Properties of Concrete.

[39] De Guzmán Báez A. (2010). Study of the Fundamental Properties of Prefabricated Non-Structural Concrete Elements Incorporating Recycled Aggregate in Their Coarse and Fine Fraction.

[40] Poon C.S., Kou S.C., Lam L. (2002). Use of recycled aggregates in molded concrete bricks and blocks. Constr. Build. Mater..

[41] Poon C., Shui Z., Lam L. (2004). Effect of microstructure of ITZ on compressive strength of concrete prepared with recycled aggregates. Constr. Build. Mater..

[42] Poon C.S., Shui Z.H., Lam L., Fok H., Kou S.C. (2004). Influence of moisture states of natural and recycled aggregates on the slump and compressive strength of concrete. Cem. Concr. Res..

[43] EN 127340 (2006). Concrete Kerb Units. Requirements and Test Methods.

[44] EN 12390-1 (2012). Testing Hardened Concrete—Part 1: Shape, Dimensions and Other Requirements for Specimens and Moulds.

[45] EN 12390-2 (2009). Testing Hardened Concrete—Part 2: Making and Curing Specimens for Strength Tests.

[46] EN 12350-3 (2009). Testing Fresh Concrete—Part 3: Vebe Test.

[47] EN 12350-1 (2009). Testing Fresh Concrete—Part 1: Sampling.

[48] EN 12390-7 (2009). Testing Hardened Concrete—Part 7: Density of Hardened Concrete.

[49] EN 1340 (2003). Concrete Kerb Units—Requirements and Test Methods.

[50] EN 1340/AC (2006). Concrete Kerb Units. Requirements and Test Methods.

[51] EN 1338 (2003). Concrete Paving Blocks—Requirements and Test Methods.

[52] EN 12390-3/AC (2011). Testing Hardened Concrete—Part 3: Compressive Strength of Test Specimens.

[53] EN 12390-4 (2000). Testing Hardened Concrete—Part 4: Compressive Strength—Specification for Testing Machines.

[54] EN 83988-1 (2008). Concrete Durability. Test Methods. Determination of the Electrical Resistivity. Part 1: Direct Test (Reference Method).

[55] Xiao Z., Ling T.C., Kou S.C., Wang Q., Poon C.S. (2011). Use of wastes derived from earthquakes for the production of concrete masonry partition wall blocks. Waste Manag..

[56] Medina C., Zhu W., Howind T., Frías M., Sánchez de Rojas M.I. (2015). Effect of the constituents (asphalt, clay materials, floating particles and fines) of construction and demolition waste on the properties of recycled concretes. Constr. Build. Mater..

[57] Medina C., Zhu W., Howind T., Sánchez de Rojas M.I., Frías M. (2014). Influence of mixed recycled aggregate on the physical—Mechanical properties of recycled concrete. J. Clean. Prod..

[58] Rodríguez-Robles D., García-González J., Juan-Valdés A., Morán-del Pozo J.M., Guerra-Romero M.I. (2015). Effect of mixed recycled aggregates on mechanical properties of recycled concrete. Mag. Concr. Res..

[59] Gonzalez-Corominas A., Etxeberria M. (2014). Properties of high performance concrete made with recycled fine ceramic and coarse mixed aggregates. Constr. Build. Mater..

[60] Shaikh F.U.A., Nguyen H.L. (2013). Properties of concrete containing recycled construction and demolition wastes as coarse aggregates. J. Sustain. Cem. Mater..

[61] Özalp F., Yılmaz H.D., Kara M., Kaya Ö., Şahin A. (2016). Effects of recycled aggregates from construction and demolition wastes on mechanical and permeability properties of paving stone, kerb and concrete pipes. Constr. Build. Mater..

[62] Sidorova A., Vazquez-Ramonich E., Barra-Bizinotto M., Roa-Rovira J.J., Jimenez-Pique E. (2014). Study of the recycled aggregates nature’s influence on the aggregate–cement paste interface and ITZ. Constr. Build. Mater..

[63] Tam V.W.Y., Gao X.F., Tam C.M. (2004). Microstructural analysis of recycled aggregate concrete produced from two-stage mixing approach. Cem. Concr. Res..

[64] Ferreira L., De Brito J., Barra M. (2011). Influence of the pre-saturation of recycled coarse concrete aggregates on concrete properties. Mag. Concr. Res..

[65] Mefteh H., Kebaïli O., Oucief H., Berredjem L., Arabi N. (2013). Influence of moisture conditioning of recycled aggregates on the properties of fresh and hardened concrete. J. Clean. Prod..

[66] Binici H., Arocena J., Kapur S., Aksogan O., Kaplan H. (2009). Microstructure of red brick dust and ground basaltic pumice blended cement mortars exposed to magnesium sulphate solutions. Can. J. Civ. Eng..

[67] Henocq P., Samson E., Marchand J. (2012). Portlandite content and ionic transport properties of hydrated C3S pastes. Cem. Concr. Res..

[68] Lee K.-H., Yang K.-H. (2016). Development of a neutral cementitious material to promote vegetation concrete. Constr. Build. Mater..

[69] Poon C.-S., Kou S., Wan H., Etxeberria M. (2009). Properties of concrete blocks prepared with low grade recycled aggregates. Waste Manag..

[70] Rigo da Silva C.A., Pedrosa Reis R.J., Soares Lameiras F., Vasconcelos W.L. (2002). Carbonation-Related Microstructural Changes in Long-Term Durability Concrete. Mater. Res..

[71] Hewlett P. (2003). Lea’s Chemistry of Cement and Concrete.

[72] Malhotra V.M., Mehta P.K. (1996). Puzzolanic and Cementitious Materials.

[73] Mindess S., Young J.F., Darwing D. (2003). Concrete.

[74] Gómez-Soberón J.M.V. (2002). Porosity of recycled concrete with substitution of recycled concrete aggregate: An experimental study. Cem. Concr. Res..

[75] Bermejo E.B., Moragues A., Gálvez J.C., Fernández Cánovas M. (2010). Permeability and pore size distribution in medium strength self-compacting concrete. Materiales de Construcción.

[76] Kumar R., Bhattacharjee B. (2003). Porosity, pore size distribution and in situ strength of concrete. Cem. Concr. Res..

[77] Moon D.J., Moon H.Y. (2002). Effect of Pore Size Distribution on the Qualities of Recycled Aggregate Concrete. KSCE J. Civ. Eng..

[78] Cortas R., Rozière E., Staquet S., Hamami A., Loukili A., Delplancke-Ogletree M.-P. (2014). Effect of the water saturation of aggregates on the shrinkage induced cracking risk of concrete at early age. Cem. Concr. Compos..

[79] Buyle-Bodin F., Hadjieva-Zaharieva R. (2002). Influence of industrially produced recycled aggregates on flow properties of concrete. Mater. Struct..

[80] Kou S.-C., Poon C.-S., Etxeberria M. (2011). Influence of recycled aggregates on long term mechanical properties and pore size distribution of concrete. Cem. Concr. Compos..

[81] Rübner K., Kühne H.C. (2008). Pore Structure of Concrete with Recycing Aggregates. 33o International Geologogical Congress.

[82] Guo Y., Qian J., Wang X. (2013). Pore Structure and Influence of Recycled Aggregate Concrete on Drying Shrinkage. Math. Probl. Eng..

[83] Medina C., Frías M., Sánchez de Rojas M.I. (2012). Microstructure and properties of recycled concretes using ceramic sanitary ware industry waste as coarse aggregate. Constr. Build. Mater..

[84] Soutsos M.N., Tang K., Millard S.G. (2012). The use of recycled demolition aggregate in precast concrete products—Phase III: Concrete pavement flags. Constr. Build. Mater..

[85] Olorunsogo F., Padayachee N. (2002). Performance of recycled aggregate concrete monitored by durability indexes. Cem. Concr. Res..

[86] Wirquin E., Hadjieva-Zaharieva R., Buyle-Bodin F. (2000). Use of water absorption by concrete as a criterion of the durability of concrete-application to recycled aggregated concrete. Mater. Struct..

[87] Pacheco-Torgal F., Jalali S. (2011). Compressive strength and durability properties of ceramic wastes based concrete. Mater. Struct..

[88] Gencel O., Ozel C., Koksal F., Erdogmus E., Martínez-Barrera G., Brostow W. (2012). Properties of concrete paving blocks made with waste marble. J. Clean. Prod..

[89] Sadek D.M., El Nouhy H.A. (2014). Properties of paving units incorporating crushed ceramic. HBRC J..

[90] Mas B., Cladera A., del Olmo T., Pitarch F. (2012). Influence of the amount of mixed recycled aggregates on the properties of concrete for non-structural use. Constr. Build. Mater..

[91] De Brito J., Pereira A.S., Correia J.R. (2005). Mechanical behaviour of non-structural concrete made with recycled ceramic aggregates. Cem. Concr. Compos..

[92] Al-Assadi G., Casati M.J., Fernández J., Gálvez J.C. (2009). Evaluación del deterioro del hormigón sometido a ciclos de hielo-deshielo. An. Mec. Fract..

[93] Bektas F., Wang K., Ceylan H. (2009). Effects of crushed clay brick aggregate on mortar durability. Constr. Build. Mater..

[94] Foy C., Pigeon M., Banthia N. (1988). Freeze-thaw durability and deicer salt scaling resistance of a 0.25 water-cement ratio concrete. Cem. Concr. Res..

[95] Pigeon M., Marchand J., Pleau R. (1996). Frost resistant concrete. Constr. Build. Mater..

[96] EN 1339 (2004). Concrete Paving Flags—Requirements and Test Methods.

[97] Salem R.M., Burdette E.G., Jackson N.M. (2003). Resistance to freezing and thawing of recycled aggregate concrete. ACI Mater. J..

[98] Zaharieva R., Buyle-Bodin F., Wirquin E. (2004). Frost resistance of recycled aggregate concrete. Cem. Concr. Res..

[99] Ghafoori N., Mathis R. (1998). Prediction of Freezing and Thawing Durability of Concrete Paving Blocks. J. Mater. Civ. Eng..

[100] Salem R.M., Burdette E.G. (1998). Role of chemical and mineral admixtures on physical properties and frost-resistance of recycled aggregate concrete. ACI Mater. J..

[101] Richardson A., Coventry K., Edmondson V., Dias E. (2016). Crumb rubber used in concrete to provide freeze–thaw protection (optimal particle size). J. Clean. Prod..

[102] Nehdi M.L., Najjar M.F., Soliman A.M., Azabi T.M. (2017). Novel eco-efficient Two-Stage Concrete incorporating high volume recycled content for sustainable pavement construction. Constr. Build. Mater..

[103] Puthussery J.V., Kumar R., Garg A. (2017). Evaluation of recycled concrete aggregates for their suitability in construction activities: An experimental study. Waste Manag..

[104] Beaty A.N.S., Raymond G.P. (1995). Concrete block road paving. Concr. Int..

[105] Hou T.C., Nguyen V.K., Su Y.M., Chen Y.R., Chen P.J. (2017). Effects of coarse aggregates on the electrical resistivity of Portland cement concrete. Constr. Build. Mater..

[106] Arredondo-Rea S.P., Corral-Higuera R., Neri-Flores M.A., Gomez-Soberon J.M., Almeraya-Calderon F., Castorena-Gonzalez J.H., Almaral-Sanchez J.L. (2011). Electrochemical Corrosion and Electrical Resistivity of Reinforced Recycled Aggregate Concrete. Int. J. Electrochem. Sci..

[107] Gonzalez A., Miren E. (2014). Experimental Analysis of Properties of High Performance Recycled Aggregate Concrete. Constr. Build. Mater..

[108] Medina C., Sánchez de Rojas M.I., Thomas C., Polanco J.A., Frías M. (2016). Durability of Recycled Concrete Made with Recycled Ceramic Sanitary Ware Aggregate. Inter-Indicator Relationships. Constr. Build. Mater..

[109] Portella K.F., Joukoski A., Franck R., Derksen R. (2006). Secondary recycling of electrical insulator porcelain waste in Portland concrete structures: DETERMINATION of the performance under accelerated aging. Cerâmica.

[110] Whittington H.W., McCarter J., Forde M.C. (1981). The conduction of electricity through concrete. Mag. Concr. Res..

[111] Mas M.A., Monzó J., Payá J., Reig L., Borrachero M.V. (2016). Ceramic tiles waste as replacement material in Portland cement. Adv. Cem. Res..

